# Gastrorenal shunt: a cause of hyperammonemia

**DOI:** 10.1002/ccr3.1323

**Published:** 2017-12-15

**Authors:** Yuya Nakamura, Isao Ohsawa, Yoshikazu Goto, Hiromichi Gotoh

**Affiliations:** ^1^ Department of Internal Medicine Saiyu Soka Hospital Soka City Saitama‐ken Japan; ^2^ Department of Pharmacology School of Medicine Showa University Shinagawa‐ku Tokyo Japan

**Keywords:** Chronic kidney disease, gastrorenal shunt, hyperammonemia

## Abstract

Gastrorenal shunts may induce hyperammonemia. Portosystemic shunts should be suspected when hyperammonemia occurs in patients with chronic kidney disease.

Clinical Question: What is the cause of hyperammonemia in this patient?

A 72‐year‐old male patient presented with sudden loss of consciousness and hyperammonemia, with blood ammonia levels that were much higher than the levels 2 days prior (increase from 129 to 340 *μ*g/dL). Because of diabetes, chronic heart failure, and chronic kidney disease (CKD) (creatinine, 4.35 mg/dL), he developed fluid overload. However, he had no history of abdominal surgery or liver dysfunction. He was given a laxative, and a solution containing branched‐chain amino acids was administered. His level of consciousness improved after excess fluid, and solutes were removed by hemodialysis (HD). Abdominal‐enhanced computed tomography revealed a gastrorenal shunt (Fig. 1).

**Figure 1:**
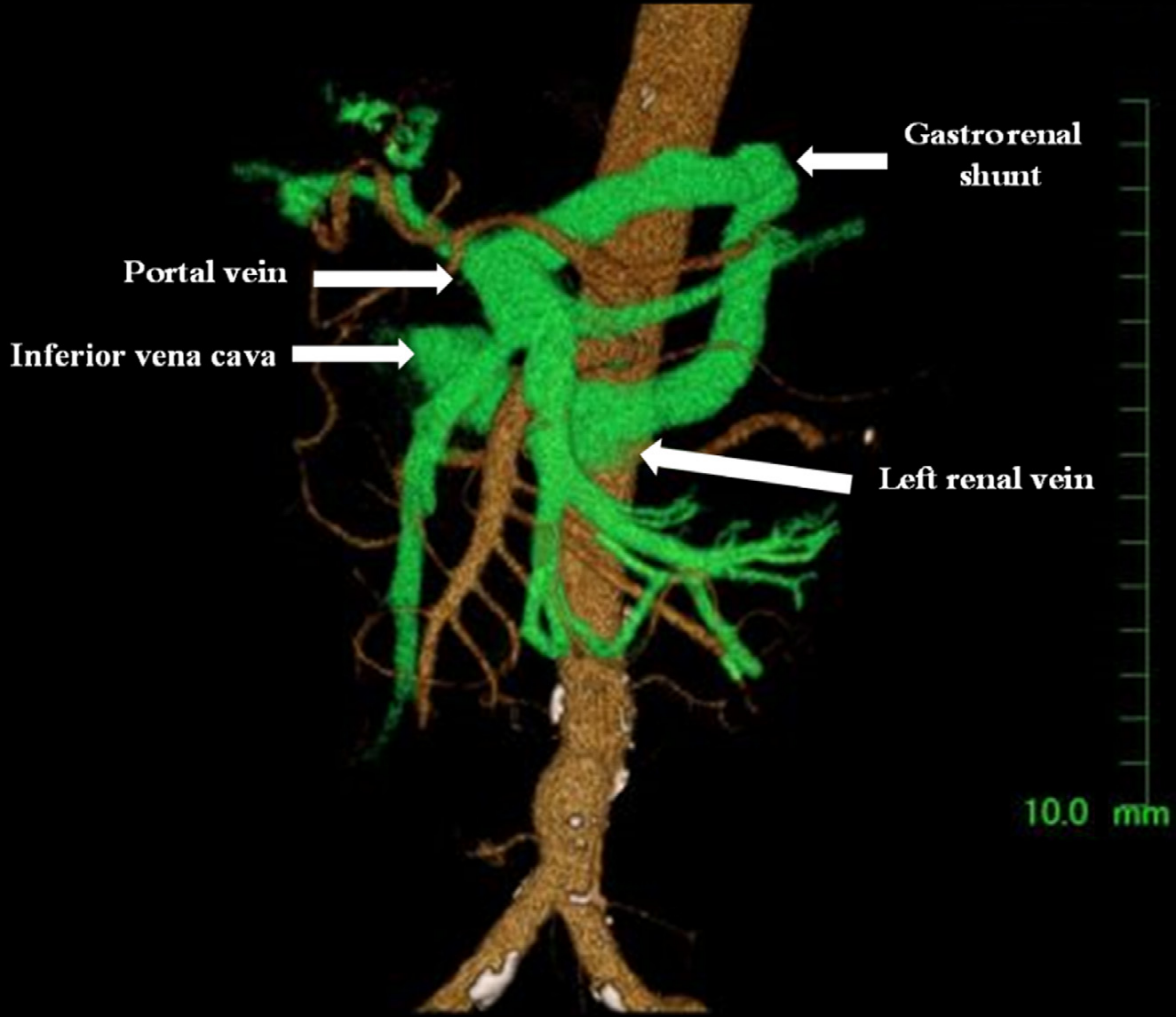
Gastrorenal shunt: Three‐dimensional reconstruction of abdominal‐enhanced computed tomography image.

In patients on maintenance hemodialysis, a gastrorenal portosystemic shunt can easily develop due to fluid overload and increased backflow with hemodialysis‐related fluid removal 1. However, there is little information on hyperammonemia induced by gastrorenal shunting in patients with CKD in the absence of HD treatment. This case emphasizes the need to suspect portosystemic shunting when hyperammonemia occurs in patients with CKD.

## Authorship

YN: wrote the first draft of the manuscript. IO: revised the manuscript critically, made critical revisions, and approved the final version. YG and HG: developed the structure and arguments jointly for the paper. All the authors reviewed and conceded the final version of the manuscript.

## Conflict of Interest

None declared.
